# XIAP 3′-untranslated region as a ceRNA promotes FSCN1 function in inducing the progression of breast cancer by binding endogenous miR-29a-5p

**DOI:** 10.18632/oncotarget.15159

**Published:** 2017-02-07

**Authors:** Qiang Wu, Hong Yan, Si-Qi Tao, Xiao-Nan Wang, Lang Mou, Ping Chen, Xing-Wang Cheng, Wen-Yong Wu, Zheng-Sheng Wu

**Affiliations:** ^1^ Department of Pathology, The Second Affiliated Hospital of Anhui Medical University, Hefei, China; ^2^ Department of Pathology, Anhui Medical University, Hefei, Anhui, China; ^3^ Department of Pathology, Anhui Provincial Cancer Hospital, Hefei, Anhui, China; ^4^ Laboratory of Pathogenic Microbiology and Immunology, Anhui Medical University, Hefei, Anhui, China; ^5^ Department of Emergency, The First Affiliated Hospital of Bengbu Medical University, Bengbu, Anhui, China; ^6^ Department of General Surgery, The First Affiliated Hospital of Anhui Medical University, Hefei, Anhui, China

**Keywords:** XIAP, 3′, UTR, miRNA, breast cancer

## Abstract

The non-coding 3′-untranslated region (UTR) of genes play an important role in the regulation of microRNA (miRNA) functions, since it can bind and inactivate multiple miRNAs. Herein, we report that ectopic expression of XIAP 3′UTR increased human breast cancer cells proliferation, colony formation, migration, invasion and xenograft tumor growth and suppressed tumor cell death. To investigate this process, we further correlated the genome-wide transcriptional profiling with the gene expression alterations after transfecting XIAP 3′UTR in MCF-7 cells. We identified a robust, genome-wide mechanism of cell migration, motility and epithelial to mesenchymal transition by which mediated by a previously described cellular component movement factor FSCN1. Expression of XIAP and FSCN1 were up-regulated synergistically after transfecting XIAP 3′UTR *in vitro* and *in vivo*. Interactions between XIAP and FSCN1 appear to be a key determinant of these processes. Co-transfection with Dicer siRNA reversed the XIAP 3′UTR-mediated oncogenicity, suggesting the miRNAs might be involved in that process. Furthermore, we demonstrated that one miRNA, miR-29a-5p, can bind to both the XIAP and FSCN1 3′UTRs and play an important role in that interactions. We showed that the 3′UTR of XIAP was able to antagonize miR-29a-5p, and resulted in the increased translation of XIAP and FSCN1. Thus, our findings reveal important new insights into how XIAP 3′UTR works, suggesting that the non-coding XIAP 3′UTR serves as a competitor for miRNA binding and subsequently inactivates miRNA functions, by which XIAP 3′UTR frees the target mRNAs from being repressed.

## INTRODUCTION

The X-linked inhibitor of apoptosis protein (XIAP), a member of inhibitors of apoptosis (IAP) family, is a essential regulator of apoptosis [1]. XIAP has demonstrated to have the most potent anti-apoptotic capacity by directly binding to caspases and inhibiting their activity [2]. Altered expression of XIAP has been shown in different kinds of human pathogenesis [1]; loss of XIAP was reported to increase the sensitivity of cells to death [3], while upregulation of XIAP is revealed in many human cancers and associated with chemical or radiation resistance [4–6]. In addition, our recent study demonstrated that XIAP was able to suppress autophagy pathway, independent its role of anti-apoptosis ability [7]. Recent studies indicated that expression of XIAP can be regulated by microRNAs (miRNA) [8–11].

MiRNA is a class of non-coding RNA molecules of approximately 22 nucleotides, which negatively regulates expression of target gene by binding to the 3′-untranslated region (UTR) at the post-transcriptional level [12]. By repressing mRNA translation, miRNAs have been reported to play an important role in physiological and pathological conditions [13]. Furthermore, the disruption of miRNAs expression has been involved in more and more diseases, including cancer [14, 15]. Recent studies have confirmed that the 3′UTR is an important target site of miRNA. Competitive endogenous RNAs (ceRNAs) form a complex regulatory network by sharing one or more miRNA response elements (MREs), which compete with miRNA target sequences for common miRNAs [16]. On the other hand, the expression of 3′UTRs could regulate the function of endogenous miRNAs [17–19]. For instance, PTENP1, which is highly homologous to the tumor suppressor gene PTEN and functions as a ceRNA to regulate the expression of PTEN by miR-21 [20]. Zina et al. demonstrated that the CD44 3′UTR serves as a CDC42 ceRNA and inhibits proliferation, colony formation, tumor growth by arresting miRNA function in breast cancer cells [17]. And Versican 3′UTR plays critical role in hepatocellular cancer progression by regulating miRNA activity [21]. Based on the above theory, we thought about the human gene XIAP which has a long 3′UTR, and whether its 3′UTR can regulate miRNA function as a ceRNA in breast cancer cells remains unclear.

In the present study, we found that XIAP 3′UTR is a tumor promoter in breast cancer cells *in vitro* and *in vivo* functional assays. In addition, our data demonstrated that XIAP 3′UTR may act as a ceRNA to regulate the expression of FSCN1 by arresting miR-29a-5p function. Therefore, our findings provided novel perspectives into the understanding of the development and progression of breast cancer.

## RESULTS

### Discrepancy between mRNA and protein expression of XIAP in serum starvation

**Figure 1:**
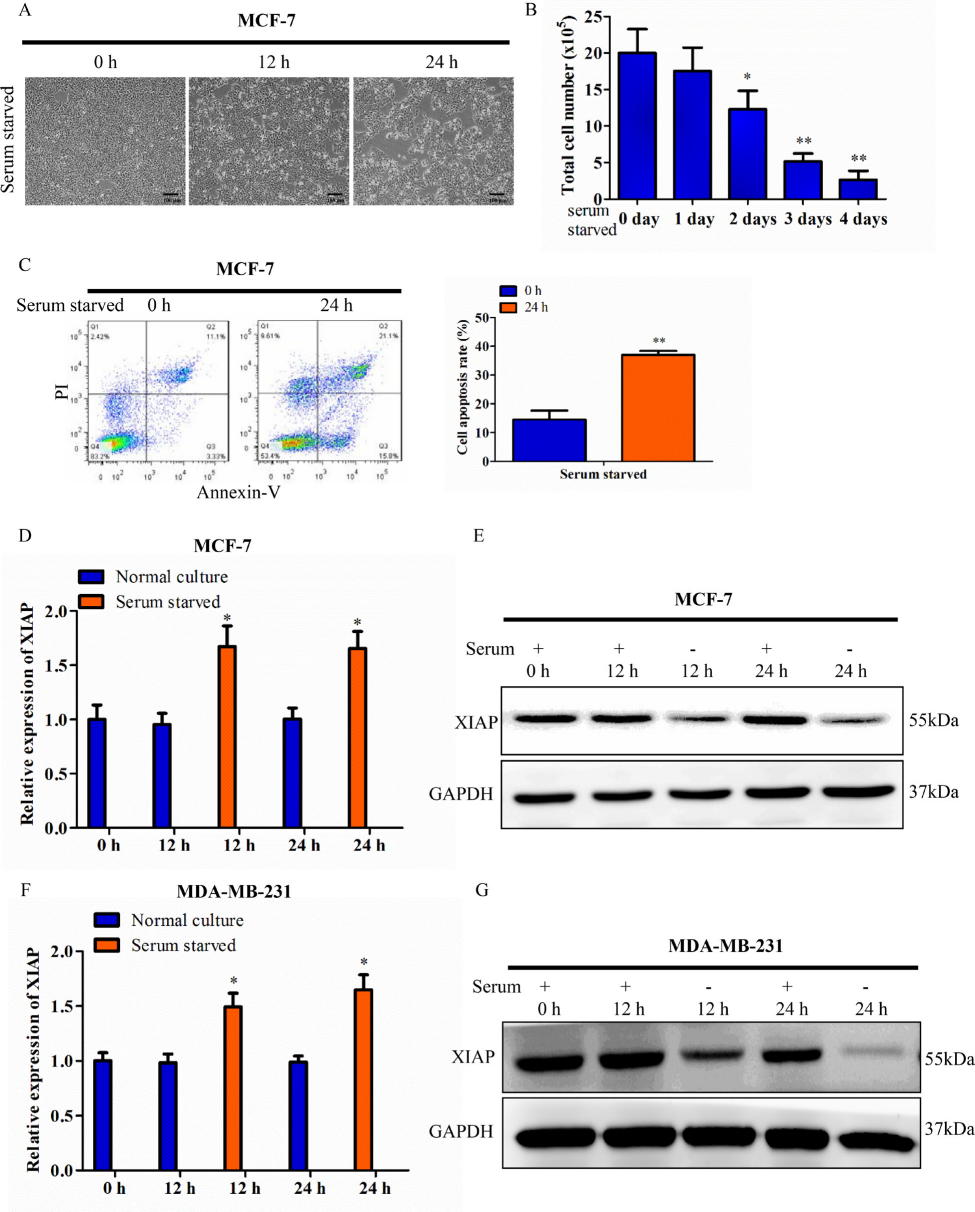
Discrepancy between XIAP mRNA and protein under serum starvation (**A**) MCF-7 cells were maintained in tissue culture dishes in serum-free conditions. Cell survival was monitored by light microscopy and photograph. Scale bar, 100 μm. (**B**) Surviving cells were harvested and counted. (**C**) MCF-7 cells were cultured in serum-free conditions for 24 h. The cells were stained with Annexin V and PI and analyzed by flow cytometry. (**D**–**E**) XIAP expression levels was checked at the transcriptional level by qRT-PCR and western blot. MCF-7 cells were cultured in medium containing 10% FBS (control) or serum starved condition for 12 h or 24 h. (**F**–**G**) XIAP expression levels in MDA-MB-231 cells under the condition of 10% FBS (control) or serum deficiency for 12 h or 24 h.

The effect of serum starvation on cell survival was determined when MCF-7 cells were maintained under serum-free conditions for 12 h or 24 h. As expected, the cell viability of MCF-7 cells was decreased significantly. Surviving cells were counted and statistically analyzed (Figure 1A–1B). Cells were stained with annexin V and PI and analyzed by flow cytometry. MCF-7 cells deprived of serum possessed more of the apoptotic cell populations compared with cells cultured in 10% serum containing conditions (Figure 1C). There is ample evidence that XIAP plays an important role in the process of apoptosis. Next, we evaluated the effect of serum starvation-induced apoptosis on XIAP expression in breast cancer cells. qRT-PCR analysis showed that the mRNA level of XIAP was significantly increased at both 12 h and 24 h in response to serum starvation compared with controls (in the presence of serum for each time point) in MCF-7 cells (Figure 1D). In contrast, western blot analysis showed that the protein level of XIAP was decreased at both 12 h and 24 h in serum-free medium (Figure 1E). Similar results were obtained in MDA-MB-231 mammary carcinoma cells and HCT116 colon carcinoma cells (Figure 1F–1G and Supplementary Figure 1A–1E). These data showed discrepant expression between XIAP mRNA and protein under conditions of serum starvation, suggesting translational regulation might be involved in that process.

To further verify this finding, we then chose a normal human mammary epithelial cell line (HMEC) and five breast cancer lines (MCF-7, MDA-MB-231, BT549, SKBR3 and T47D) to assess the roles of XIAP 3′UTR using qRT-PCR. We found that XIAP 3′UTR mRNA levels were significantly higher in breast cancer cells than in normal mammary epidermal cells (Supplementary Figure 1F). Accordingly, while MCF-7 and MDA-MB-231 cells in serum starvation culturing condition, mRNA level of XIAP 3′UTR was significantly increased at both 12 h and 24 h compared with controls in serum containing culturing condition (Supplementary Figure 1G–1H).

### Expression of XIAP 3′UTR promoted proliferation, survival, migration and invasion of breast cancer cells *in vitro*

An expression plasmid was constructed to study the function of the XIAP 3′UTR. The conserved region of the XIAP 3′UTR (6798bp, Genebank access number, NM_001204401) was cloned into pIRESneo3 vector (Promega) to generate the XIAP 3′UTR plasmid (Supplementary Figure 2A). To determine the role of XIAP 3′UTR, we forced the expression of XIAP 3′UTR in breast cancer cells. Initially, the vector harboring XIAP 3′UTR was introduced into MCF-7 and MDA-MB-231 cells to produce two stable cell lines respectively. After that, we confirmed elevated XIAP 3′UTR expression in MCF-7-XIAP 3′UTR and MDA-MB-231-XIAP 3′UTR cells, respectively (Supplementary Figure 2B). In addition, breast cancer cell lines BT549, SKBR3 and T47D were also transiently transfected with the pIRES-XIAP 3′UTR expressing construct. Control cells were established with use of the empty pIRES vector. XIAP 3′UTR plasmid transfected cells showed higher levels of XIAP 3′UTR than the control group (Supplementary Figure 2C–2E).

**Figure 2:**
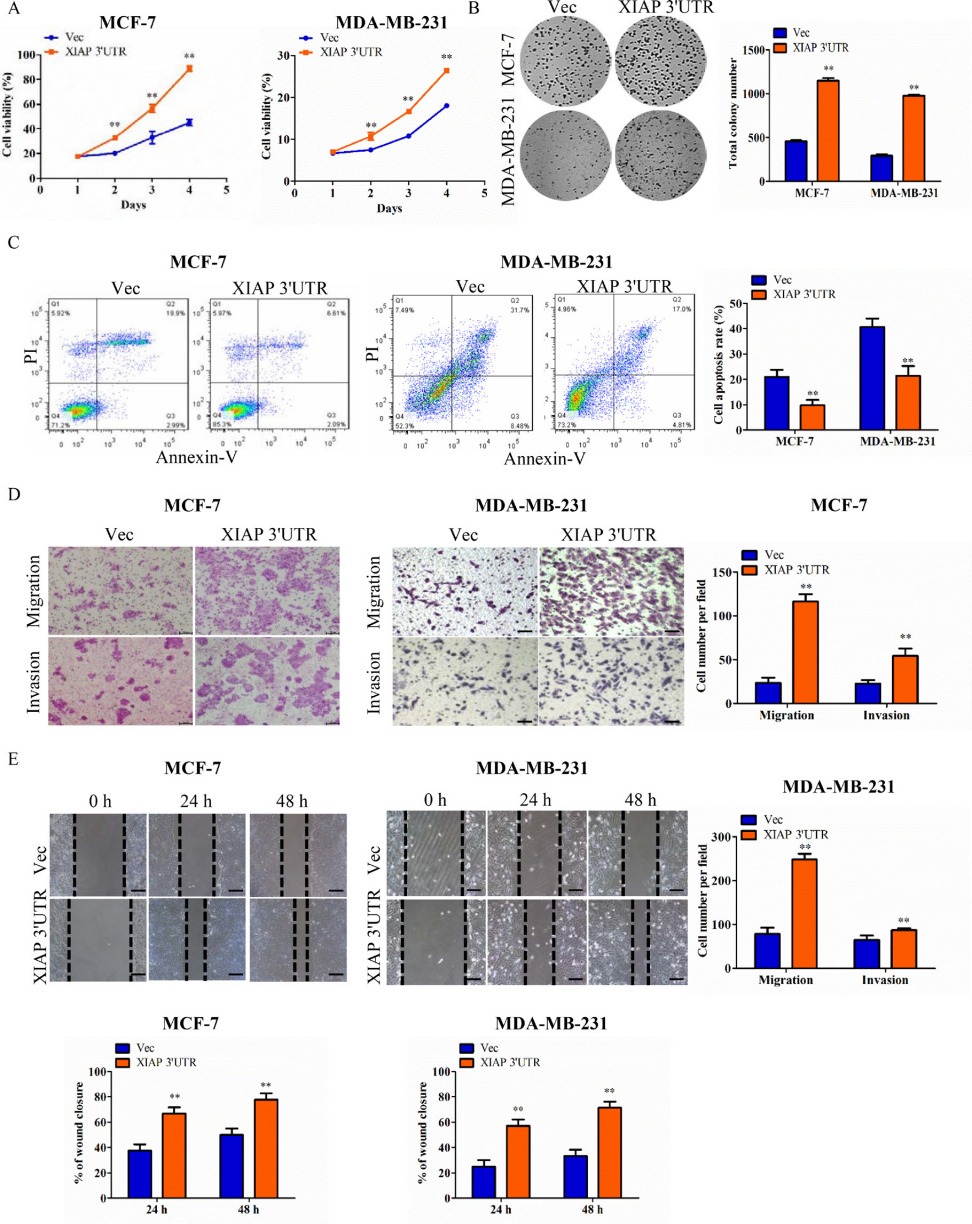
Functional characterization of XIAP 3′UTR in breast cancer cells *in vitro* (**A**) Cells were seeded in 96-well plates (2 × 10^3^ per well) and grown for 5 days for MTT assay. Cell viability MTT assays in MCF-7 cells (left) or MDA-MB-231 cells (right). (**B**) Colony formation of vector and XIAP 3′UTR construct in MCF-7 or MDA-MB-231 cells. Total colonies numbers were shown on the right panel. (**C**) XIAP 3′UTR and vector-transfected cells were cultured for apoptosis assay. After being stained with Annexin V and PI, the cells were analyzed by flow cytometry. The XIAP 3′UTR-transfected cells displayed lower level of apoptotic and necrotic cells compared with the control cells. Percentages of apoptosis cells were calculated. (**D**) Tumor cell migration and invasion assay. MCF-7cells or MDA-MB-231 cells were grown and stably transfected with XIAP 3′UTR or negative control. Cells in the upper chamber were removed and those cells migrated to the lower layer of the inner chamber were stained and counted. Scale bar, 100 μm. (**E**) Wound healing assay. Wounded areas were examined under ×100 magnification. Scale bar, 100 μm. **P < 0.01.

MCF-7, MDA-MB-231 and HCT116 cells stably transfected with XIAP 3′UTR were subjected to a series of cell function assays. In cell viability assays, the proliferation rates of XIAP 3′UTR transfected cells were significantly higher than that of the controls (Figure 2A; Supplementary Figure 2F–2H and Supplementary Figure 3A). In colony formation assays, the cells with stably transfected XIAP 3′UTR formed larger and more numerous colonies per plate than those in the control cells (Figure 2B and Supplementary Figure 3B). In apoptosis assays, the XIAP 3′UTR expressing cells exhibited significantly decreased rates of apoptosis (Figure 2C). By use of transwell assays, the cells with forced expression of XIAP 3′UTR exhibited increased rates of migration and invasion compared with the control (Figure 2D and Supplementary Figure 3C). Furthermore, wound healing assays demonstrated that forced expression of XIAP 3′UTR in cells led to faster closing of the scratch wounds compared with the control cells (Figure 2E).

### XIAP 3′UTR expression level was associated with EMT features of breast cancer

**Figure 3:**
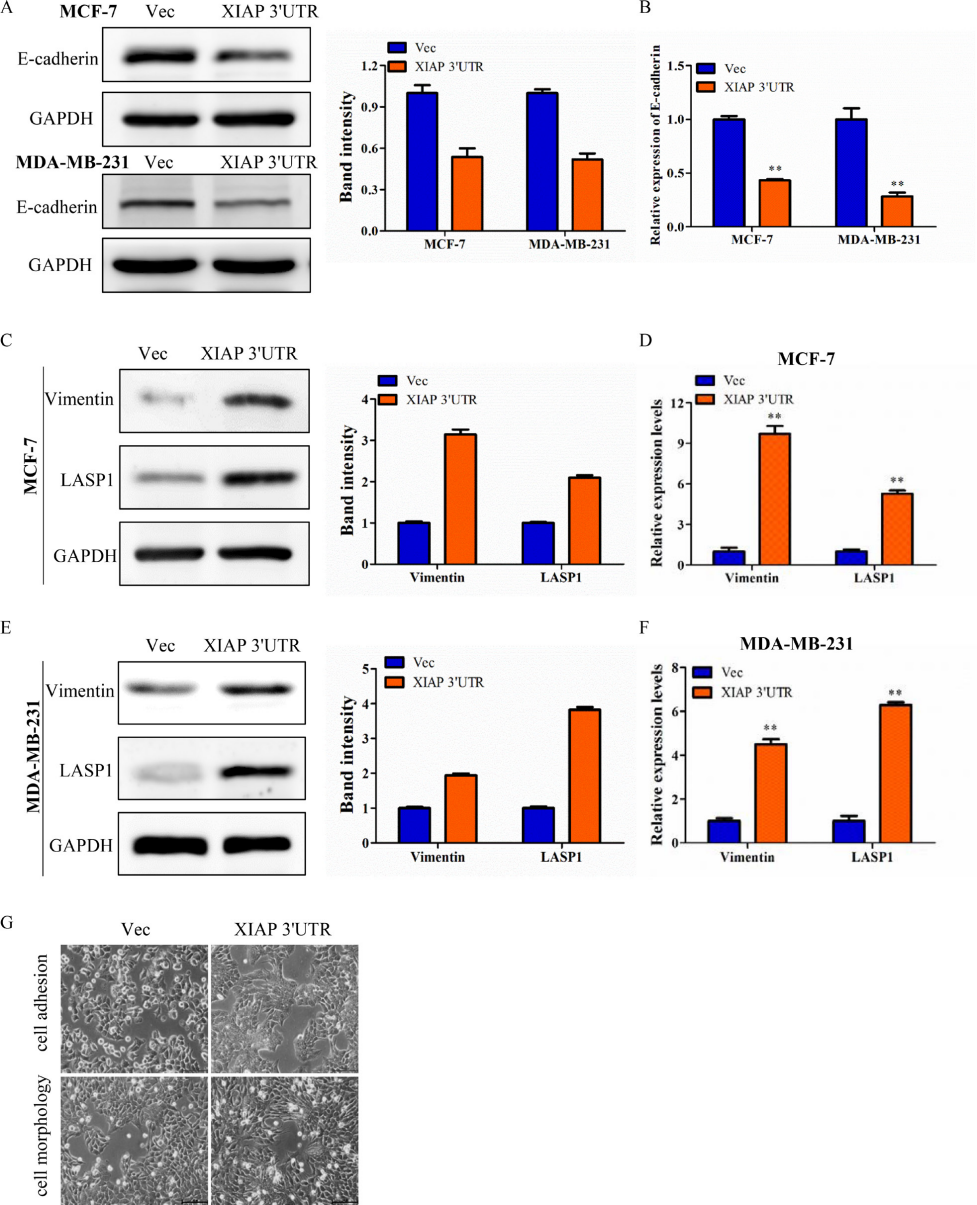
XIAP 3′UTR was associated with EMT features of breast cancer (**A**) Western blot analysis of MCF-7 and MDA-MB-231 for E-cadherin protein expression after transfection with XIAP 3′UTR. The relative expression of E-cadherin was normalized to GAPDH. (**B**) qRT-PCR analysis of E-cadherin in XIAP 3′UTR-treated MCF-7 and MDA-MB-231 cells. (**C**–**F**) Expression of Vimentin and LASP1 by western blot (left) and qRT-PCR (right) with overexpression of XIAP 3′UTR. Representative quantitative data of densitometric analyses were shown in middle. (**G**) Morphology of XIAP 3′UTR cells and control cells. XIAP 3′UTR cells exhibited elongation. Representative pictures were captured using phase-contrast microscopy at 200 x magnification.

As we found high levels of XIAP 3′UTR were strongly associated with increasing capacity of metastasis in breast cancer, more and more evidence indicates that promotion of epithelial-mesenchymal transition (EMT), which refers to the transformation of epithelial cells from a well-differentiated phenotype to an invasive mesenchymal phenotype under pathological conditions [22]. To evaluate whether XIAP 3′UTR modulates EMT, we then detected the expression of epithelial and mesenchymal markers by western blot. XIAP 3′UTR transfected cells expressed lower levels of the epithelial marker (E-cadherin), and higher levels of the mesenchymal marker (Vimentin) as well as LASP1 (Figure 3A, 3C, 3E), a cytoskeletal scaffold protein that plays an important role in cytoskeletal organization and cell migration [23]. These were in consistent with prior research that molecular characterization of LASP1 expression revealed Vimentin as its new partner in human hepatocellular carcinoma cells [24]. Similar results were obtained by qRT-PCR (Figure 3B, 3D, 3F). Moreover, in two dimensional culture, XIAP 3′UTR transfected cells assumed a scattered and spindle-like morphology whereas control cells were tightly interconnected and exhibited an epithelial-like morphology, demonstrating XIAP 3′UTR may regulate breast cancer cytoskeletal dynamics which is often linked to cell motility and metastatic potential (Figure 3G). Our data suggested that a positive correlation existed between the expression of XIAP 3′UTR and some EMT features, which likely contribute to the observed aggravation of tumor invasion and metastasis of breast cancer.

### Expression of XIAP 3′UTR promoted growth and invasiveness of MCF-7 cells *in vivo*

**Figure 4:**
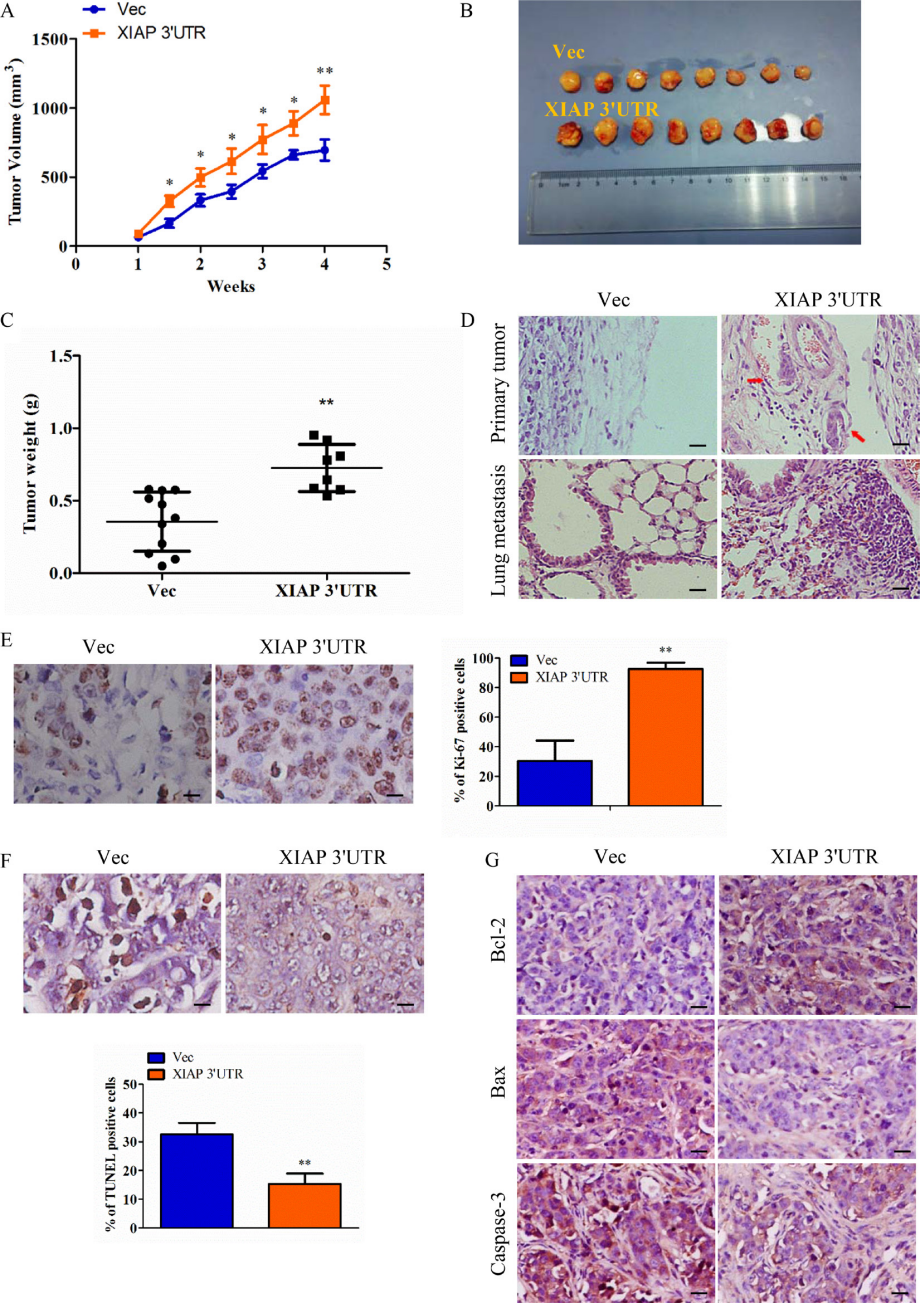
Expression of XIAP 3′UTR enhances tumor growth and metastasis *in vivo* (**A**–**B**) MCF-7 cells transfected with XIAP 3′UTR or the control vector were injected subcutaneously into BALB/c nude mice. Tumor growth curve (right) and final tumor sizes (left) was monitored for up to 4 weeks, when the tumors reached the limit sizes. (**C**) Tumor weight. The terminal tumor weight was plotted for each treatment group. Significantly higher tumor weight in mice injected with XIAP 3′UTR versus control cells. (**D**) Sections from primary tumors and lungs were examined. Tumor cell invasion is shown in the top panel and pulmonary micro-metastases are shown in the bottom panel. The arrows indicate metastatic tumor foci. Scale bar, 50 μm. (**E**–**F**) Cell proliferation as measured by Ki-67 staining and cell apoptosis as measured by TUNEL staining of tumors derived from MCF-7 XIAP 3′UTR or MCF-7 Vec cells. Scale bar, 10 μm. (**G**) IHC was used to determine the expression levels of apoptosis markers which are Bcl-2, Bax and caspase-3 in mouse orthotopic tumor tissues. Scale bar, 25 μm. *P < 0.05, **P < 0.01.

To evaluate the role of XIAP 3′UTR expression on breast cancer progression *in vivo*, the xenograft model of MCF-7-Vec and MCF-7-XIAP 3′UTR cells in female BALB/c nude mice was adopted. It is obvious that tumors generated from the MCF-7-XIAP 3′UTR group were significantly larger than those of MCF-7-Vec group (Figure 4A–4B). Primary tumor weights were measured on sacrifice (Figure 4C). Cancer metastasis refers to the invasion of cancer cells into adjacent tissues through the circulatory system, and in the end grows from micrometastases into larger secondary tumors [25]. Histology of xenografts revealed that tumors derived from MCF-7-XIAP 3′UTR cells were poorly encapsulated and highly invasive when compared to tumors generated by control cells. Interestingly, several tumor cell emboli in the blood vessel and lung metastases were observed in MCF-7-XIAP 3′UTR xenografts but not in the control xenografts, indicative that XIAP 3′UTR expression promoted tumor cell metastasis from the primary lesion (Figure 4D). Furthermore, forced expression of XIAP 3′UTR increased cell proliferation in the tumor, as determined by immunohistochemical analysis of nuclear incorporation of Ki-67 (Figure 4E), and also decreased cell apoptosis as determined by TUNEL assay (Figure 4F). For better understanding of the apoptosis efficacy of XIAP 3′UTR *in vivo*, we examined the expression of Bcl-2, Bax and Caspase-3 in tumor samples immunoreactivity. Bax and caspase-3 were inhibited by XIAP 3′UTR while Bcl-2 expression was increased (Figure 4G).

### Perturbation of gene expression in MCF-7 cells by XIAP 3′UTR transfection

**Figure 5:**
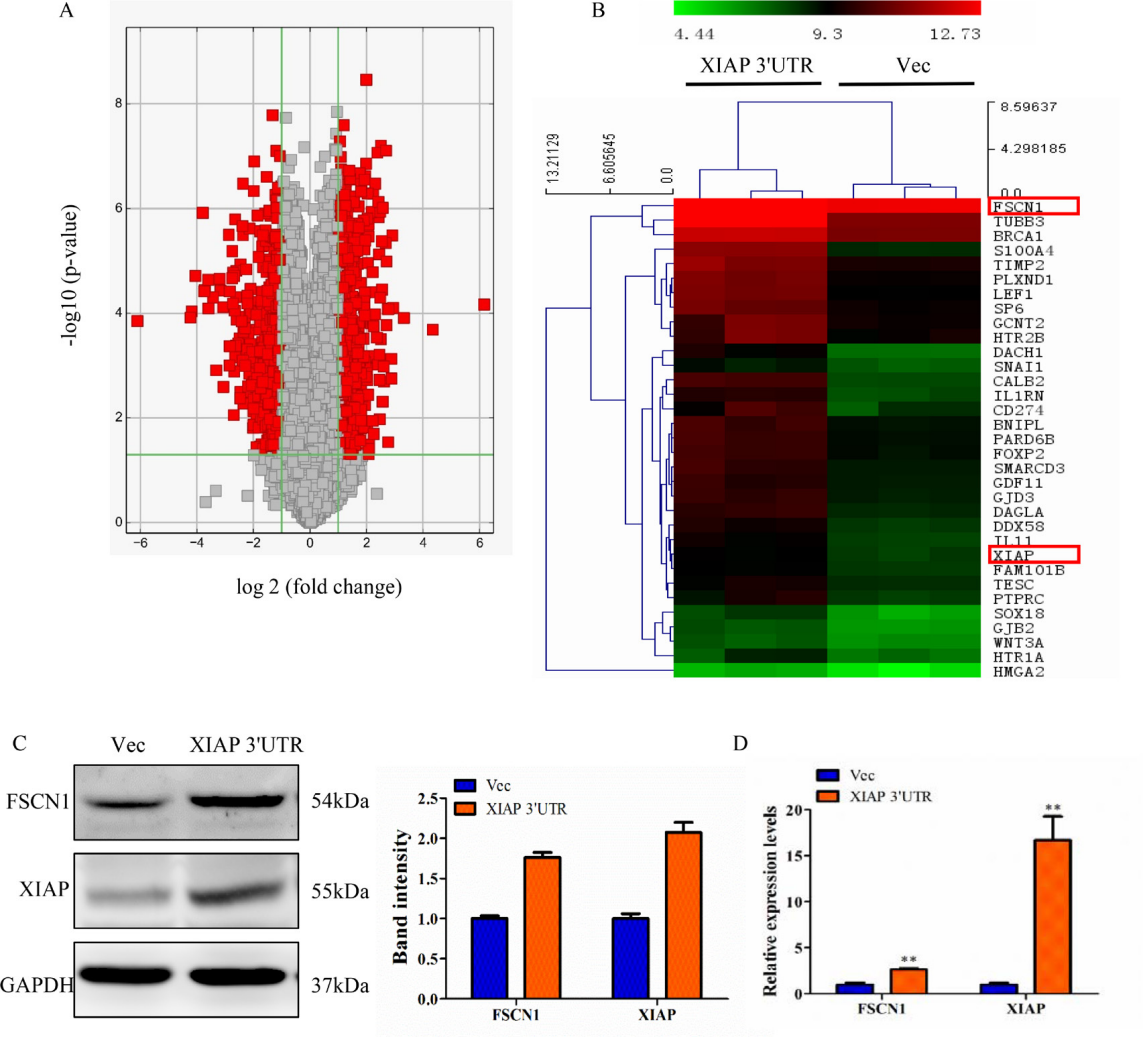
Perturbation of gene expression by XIAP 3′UTR transfection (**A**) Volcano plot representing fold change and significance of altered microarray probes in MCF-7 cells stably post-transfection with XIAP 3′UTR or vector. (**B**) Heat map depicting the mRNA expression profile of selected genes. Red squares correspond to increased expression, while green squares correspond to decreased expression of mRNA levels. (**C**) Western blot analysis of MCF-7 for FSCN1 and XIAP protein expression after stably transfection with XIAP 3′UTR. Changes of FSCN1 and XIAP band intensity relative to GAPDH intensity were shown on right. (**D**) mRNA levels of FSCN1 and XIAP were analyzed by qRT-PCR. **P < 0.01.

To better understand the mechanisms through which XIAP 3′UTR regulates breast cancer progression, we performed gene expression array analysis using RNA isolated from MCF-7 cells which were transfected with XIAP 3′UTR or vector stably. After data normalization, differentially expressed genes were assigned based on a cut-off of a fold-change of at least 2.0 and *p*-value < 0.05 (Figure 5A). Cluster analysis identified a total of 1651 genes that were significantly differentially expressed by XIAP 3′UTR transfection, relative to vector cells (Figure 5B).

To determine the mechanism of the functional effects of XIAP 3′UTR, bioinformatics methods were used to analyze the biological processes of these genes by GO or the KEGG pathway. We found 4 out of the top 11 most significantly enriched GO terms and/or pathways were related to cell motility. In addition, cell responses to cell proliferation, EMT and gap junction activity were also prominent (Supplementary Figure 4A–4B).

Next, we screened several genes known to be involved in cancer invasion and metastasis in MCF-7 cells transfected with XIAP 3′UTR or control. By a combination of these strategies, two genes attracted our attention, FSCN1 and XIAP. FSCN1, an actin-binding protein, is important for the formation of actin-based motility-structures [26]. In many human cancers including breast cancer, FSCN1 expression correlates with clinically aggressive tumors and metastasis [27]. Consistent with this, our study showed that knockdown of endogenous FSCN1 using siRNA technique greatly reduced cell migration and invasion ability in MCF-7 and MDA-MB-231 cells (Supplementary Figure 4C–4D). These results prompt us to investigate the potential relationship between FSCN1 and XIAP in breast cancer. Moreover, we found that XIAP 3′UTR-transfected breast cancer cells had significantly higher levels of FSCN1 and XIAP protein and mRNA compared to control cells (Figure 5C–5D).

### XIAP 3′UTR interacts with miRNAs and regulates expression of associated genes

**Figure 6:**
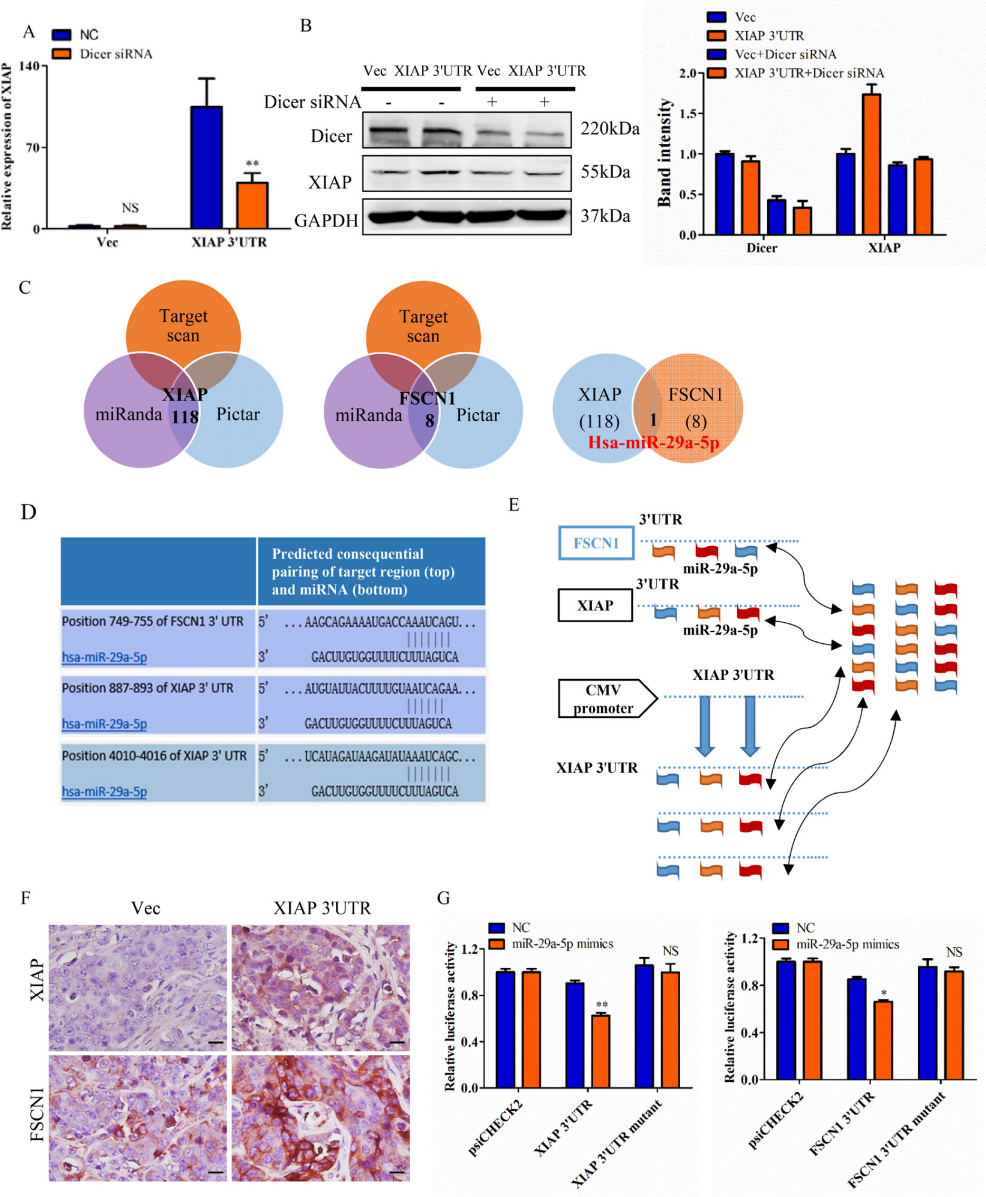
XIAP 3′UTR interacts with miRNAs and regulates expression of associated genes (**A**) MCF-7 cells were transfected with XIAP 3′UTR and/or siRNA of Dicer. mRNA level of XIAP was increased in the absence of Dicer siRNA but decreased with Dicer siRNA. (**B**) The protein level of XIAP and Dicer were detected in MCF-7 cells transfected with XIAP 3′UTR combined with or without Dicer siRNA. Representative quantitative data of densitometric analyses were shown on right. (**C**) Schema of the candidate genes predicted by different prediction methods. Each circle represents thenumber of genes identified by one algorithm. miR-29a-5p was listed in the overlap of three circles are simultaneously predicted by different algorithms. (**D**) Predicted miR-29a-5p binding sites in the 3′UTR of XIAP and FSCN1. (**E**) Hypothesis that overexpression of XIAP 3′UTR would bind endogenous miR-29a-5p and thus free XIAP and FSCN1 mRNAs for translation. (**F**) Tumor sections were immunohistochemically stained with antibody against XIAP and FSCN1. XIAP 3′UTR sections showed higher levels of these proteins than the control sections. Scale bar, 25 μm. (**G**) MCF-7 cells were co-transfected with miR-29a-5p mimics and the luciferase constructs or a mutant constructs, in which the miRNA target sites were mutated. A non-related fragment of complementary DNA was used as a positive control. *P < 0.05, **P < 0.01, NS, not significant by the Student's *t*-test.

To elucidate whether the effect of XIAP 3′UTR expression in cell proliferation dependent on miRNAs, we conducted cell lines lacking functional Dicer, a ribonuclease essential for miRNA biogenesis and whose deficiency can lead to a dramatic reduction in the levels of mature miRNAs [28]. We transfected MCF-7 cells with siRNAs targeting Dicer to block the pathway of miRNA biogenesis. And qRT-PCR was used to confirm the knockdown efficiency. Overexpression of XIAP 3′UTR significantly increased the expression of XIAP in MCF-7 cells. However, XIAP 3′UTR mediated increasing of XIAP expression was obviously lost when Dicer was knocked down. XIAP expression did not change when we cotransfected XIAP 3′UTR with Dicer siRNA (Figure 6A). The efficiency of transfection was tested by western blot in MCF-7 cells (Figure 6B). Therefore, our data implied that XIAP 3′UTR can bind to and antagonize certain endogenous miRNAs, thereby modulating the expression of XIAP.

To examine whether XIAP 3′UTR exerted its biological functions by regulating certain miRNAs which interacted with complementary sequences, we examined the potential miRNAs targeting FSCN1 and XIAP. It is known that FSCN1 is involved in cell migration, motility and invasiveness [26, 29, 30]. We employed three algorithms (TargetScan [31], miRanda [32] and the Pictar [33]) to search for the potential downstream targets and we identified the common miRNA predicted by all three programs (Figure 6C). Furthermore, we observed that miR-29a-5p possesses putative target sites on both XIAP and FSCN1 3′UTR (Figure 6C). Comparing the sequences of miR-29a-5p with XIAP and FSCN1, we found these two mRNAs were highly homologous in miRNA binding sites (Figure 6D).

We then presumed that overexpression of XIAP 3′UTR would bind and sequester miR-29a-5p, resulting in increased expression of XIAP and FSCN1 mRNA and protein levels (Figure 6E). The reason for it might be that the miR-29a-5p could bind to the exogenously transfected XIAP 3′UTR and then relive the expression of XIAP and FSCN1 (Figure 6E). This hypothesis was proved by western blot and qRT-PCR in both MDA-MB-231 and HCT116 cells (Supplementary Figure 5A–5D). Furthermore, XIAP 3′UTR and XIAP coding constructs (CDS) efficiently expression XIAP protein in HEK-293T cells, but XIAP 3′UTR constructs overexpressed XIAP compared to the XIAP CDS constructs owing to loss of miRNA-mediated suppression, whereas the XIAP CDS constructs do not (Supplementary Figure 5E). Taken together, this XIAP 3′UTR allows us to compare specifically the roles of XIAP protein and transcript function on breast cell transformation. Xenograft tumors generated with the XIAP 3′UTR-transtected MCF-7 cells were analyzed by immunostaining. It was observed that the tumors generated by MCF-7 cells with forced expression of XIAP 3′UTR tumors exhibited higher protein expression of XIAP and FSCN1, as compared with the control tumors (Figure 6F).

To verify the targeting of miR-29a-5p to both XIAP 3′UTR and FSCN1 3′UTR, human XIAP and FSCN1 3′UTR fragments containing wild-type or mutant miR-29a-5p binging sites were introduced into the downstream of the luciferase reporter gene. And the relative luciferase activity of the reporter containing wild-type XIAP and FSCN1 3′UTR were significantly inhibited when miR-29a-5p mimics was cotransfected with the reporter plasmids, whereas the luciferase activity of the reporter containing mutant 3′UTR was not affected (Figure 6G).

### miR-29a-5p level is affected in the presence of XIAP 3′UTR

**Figure 7:**
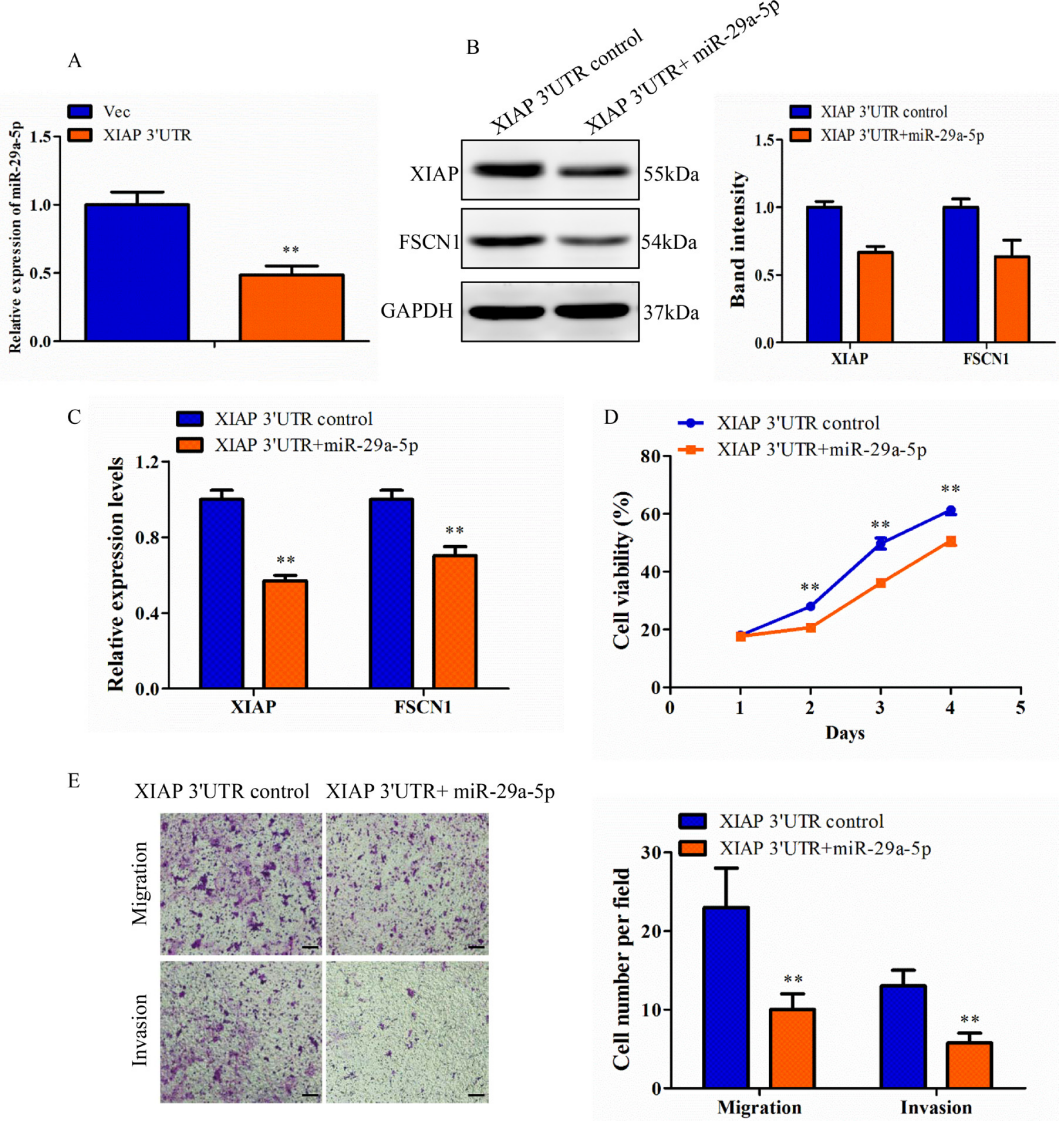
Confirmation of XIAP 3′UTR effect was regulated by miRNA in MCF-7 cell line (**A**) Total RNA from XIAP 3′UTR and vector cells were subjected to qRT-PCR analysis for expression of miR-29a-5p. (**B**) Lysates of MCF-7-XIAP 3′UTR cells transfected with NC or miR-29a-5p mimics were analyzed by western blot. Representative quantitative data of densitometric analyses were shown on right. (**C**) XIAP 3′UTR cells transfected with NC or miR-29a-5p mimics were analyzed by qRT-PCR. (**D**) The proliferation rate was measured in the XIAP 3′UTR cells transfected with miR-29a-5p mimics over a period of 4 days. (**E**) Transwell migration and invasion assay of XIAP 3′UTR cells transfected with miR-29a-5p mimics or control. Scale bar, 100 μm. **P < 0.01.

To confirm how XIAP 3′UTR affected the miRNA expression *in vitro*, we first detected the expression of miR-29a-5p in breast cancer cell lines and found that it was significantly lower than that the normal HMEC cells (Supplementary Figure 6A). Next, we chose MCF-7 cell as a model for the following gain-of-funciton and loss-of-function analysis. Expression of miR-29a-5p in MCF-7 cells transfected with miR-29a-5p mimics or inhibitor were demonstrated by qRT-PCR (Supplementary Figure 6B). In order to investigated the interaction between XIAP 3′UTR and miR-29a-5p, we assessed the interaction between the two molecules by altering the expression levels of XIAP 3′UTR and observing the effect on the expression of the other one molecule. We found that cells transfected with XIAP 3′UTR lost 51.5% expression of miR-29a-5p compared with the control cells (Figure 7A). These results suggested negative regulation between XIAP 3′UTR and miR-29a-5p.

### Validation of miR-29a-5p function

The function of miR-29a-5p relative to the XIAP 3′UTR was assessed in the MCF-7 XIAP 3′UTR cells. Western blot analysis for XIAP and FSCN1 was performed on XIAP 3′UTR cells transfected with miR-29a-5p or negative control oligonucleotides. Repression of XIAP and FSCN1 levels by the forced expression of miR-29a-5p mimics was observed as compared with the control (Figure 7B). This confirmed that the XIAP 3′UTR and FSCN1 3′UTR were both direct targets of the miR-29a-5p. Consistent with this result, qRT-PCR analysis showed that there was a decrease in the levels of both XIAP and FSCN1 mRNA, targeted by miR-29a-5p (Figure 7C). We also examined phenotypic changes in the XIAP 3′UTR cells transfected with miR-29a-5p mimics or negative control oligonucleotides, followed by measurement of cell viability. MiR-29a-5p transfected cells exhibited a decreased proliferation rate than the control cells (Figure 7D). The functions of miR-29a-5p were further investigated by migration and invasion assay. Relative to control cells, a decreased migratory and invasive capacity was observed in XIAP 3′UTR cells that were transfected with miR-29a-5p mimics (Figure 7E). In addition, it was confirmed that miR-29a-5p knockdown was able to upregulate XIAP and FSCN1 protein and mRNA levels in the control MCF-7 cells (Supplementary Figure 6C–6D). The decreased expression of miR-29a-5p indirectly identified XIAP 3′UTR function and promoted proliferation, migration and invasion in MCF-7 cells (Supplementary Figure 6E–6F). Thus, these results demonstrate XIAP 3′UTR, through its miR-29a-5p binding sites, displaces FSCN1 from miRNA-mediated repression. In total, these results indicated that the XIAP 3′UTR, is an important effector of blocking recruitment of FSCN1 to the miRNA repression complex.

## DISCUSSION

In this study, we initially detected XIAP expression under the condition of serum-free *vs*. serum-containing and found that the levels between XIAP mRNA and protein were lack of correlation in breast cancer cells, which prompted us to explore the mechanisms underlying of XIAP mRNA regulation. One point of view was regulation by miRNAs. Destabilization of miRNAs for target mRNA regulation is a major cause of reduced protein synthesis. Utilizing bioinformatics analyses, we found that XIAP mRNA 3′UTR contains a large number of putative miRNA binding sites (data not shown). Herein, we show that miR-29a-5p was able to bind the XIAP 3′UTR.

3′UTR is an important component of the ceRNA network. Therefore, changes in 3′UTRs are thought to have a significant effect on the regulation of ceRNA [16]. For instance, FOXO1 3′UTR exerts an inhibitory effect on the metastases of breast cancer cells [22]. In addition, 3′UTR shortening was observed in human cancer cells, which also affected the ceRNA network [34]. Therefore, we cloned the XIAP 3′UTR constructs into breast cancer cells. First, we demonstrated that overexpression of XIAP 3′UTR increased the proliferation rate of breast cancer cells *in vitro* and promoted tumor growth *in vivo*. It should be noted that cell proliferation only represents one aspect of tumorigenicity, and cell survival is often associated with tumorigenicity, especially under poor conditions such as in serum-free medium [17]. On the other hand, cell death also reflects the phenomenon of tumor growth. Our data indicated that the XIAP 3′UTR cells had significantly decreased rates of apoptosis. We also found that TUNEL staining observed in tumors formed by XIAP 3′UTR transfected cells resulted in a low occurrence rate of cell death. This phenomenon can be explained by the increased amount of anti-apoptotic proteins caused by XIAP 3′UTR, since increased XIAP 3′UTR levels could bind and arrest the function of endogenous miRNAs in breast cancer cells. As for the downstream anti-apoptotic effects, further studies are needed and the proteins identity involved are determined.

The biological effects of XIAP are largely due to its effect on the apoptotic pathway and have been extensively studied [7]. Recent reports demonstrated that multiple miRNAs through their down-regulation function can regulate XIAP in ovarian cancer cells, thus enhancing our understanding of the function and regulation of XIAP [35]. Considering that the 3′UTR of XIAP spans approximately 6 kb, we assume that 3′UTR may be involved in translational regulation of XIAP through the potential binding sites of miRNAs.

It is conceivable that the overexpression of XIAP 3′UTR could increase XIAP expression by modulating identical miRNAs function. This is supported by recent study showing that Versican 3′UTR expression upregulated Versican expression [19, 36]. The effect of XIAP 3′UTR on other genes expression would be much complex, as many miRNAs can bind to both 3′UTRs with high affinity. Of particular importance, our microarrays revealed an up-regulation of FSCN1 transcripts in XIAP 3′UTR cells. Several studies have reported that FSCN1 is highly expressed in malignant tumors, and is associated with increased cell motility and aggressive behavior of tumors [29, 37–39]. This is similar to the findings in breast cancer, where overexpression of FSCN1 was correlated with invasion and predicts poor survival [30]. This is in consistent with our current report that knockdown of FSCN1 expression by small interfering RNA inhibited cell migration and invasion. Up to now, genome-wide approaches based on microarray platforms have succeeded in providing extensive catalogs of differentially expressed genes, including important biomarkers for monitoring pathologic progression. Although at an early stage, the introduction of next-generation sequencing technology enhances our understanding of altered transcription mechanisms and provides new strategies for current clinical treatment [40]. For example, Mercer et al. [41] showed a discordant pattern of expression between the CDS and 3′UTR by microarray, thereby increasing the understanding of 3′UTR. Considering the novel and unexpected roles of 3′UTR-associated RNAs transcripts, the presence and different expression of 3′UTR-associated RNAs are worth considering in future studies [41]. Thus, we described novel probe-level microarray analyses to define molecular signatures.

Indeed, our data demonstrated that XIAP 3′UTR functioned as a decoy sponge binding to miRNAs, resulting in increased XIAP and FSCN1 expression levels. Inspired by above theory, we hypothesized that the XIAP 3′UTR served as a ceRNA for FSCN1 in breast cancer. When we found that miR-29a-5p can interact with both XIAP 3′UTR and FSCN1 3′UTR, we demonstrated that miRNA may play a synergistic role in regulating XIAP 3′UTR. This view is related to the concept that miRNAs with similar functions in invarious disease processes are clustered together [17]. Many miR-29 family members are reported to be dysregulated and closely related to prognosis in cancers [42]. Here, our study showed that increased levels of miR-29a-5p inhibited cell proliferation, migration and invasion, whereas knockdown of miR-29a-5p expression increased these capabilities. It seems that expression of XIAP 3′UTR have similar effect as of miR-29a-5p inhibitor. Additionally, we found that exogenous overexpression of XIAP 3′UTR could decrease miR-29a-5p levels. These results suggested that 3′UTR not only mediates transcriptional regulation by miRNAs, but also modulates miRNAs activity by binding to them. This may be another mechanism for XIAP 3′UTR inhibition of miRNA function. In this case, 3′UTR may be used to bind multiple carcinogenic miRNAs, which is one feature that can be utilized. Considering the number of miRNAs that can interact with 3′UTR, ectopic expression of 3′UTR would have a great advantage than other methods against miRNA function. This may become an ideal technology for clinical development therapeutics.

In summary, we demonstrated that XIAP 3′UTR increases cell proliferation, migration, invasion, tumor growth, EMT phenotype and decreases cell apoptosis by binding to endogenous miRNAs. In addition, on the basis of the interaction of the XIAP 3′UTR and FSCN1 3′UTR mediated by miR-29a-5p, exogenous expression of XIAP 3′UTR increased XIAP and FSCN1 expression levels. This is due to the overexpression of non-coding transcripts reduced the inhibitory effect of miRNAs on potential targets, resulting in up-regulation of multiple protein levels. By manipulating the overexpression of 3′UTRs to modulate miRNA activity, a novel approach can be used to study the function of target molecules.

## MATERIALS AND METHODS

### Cell lines and culture

Human breast cancer MCF-7, MDA-MB-231, BT549, SKBR3, T47D, human Embryonic Kidney HEK-293T and human colon cancer HCT116 cell lines were obtained from the American Type Culture Collection (ATCC, Manassas, VA, USA) and cultured under the ATCC-recommended conditions. For serum starvation, cells were initially grown in medium supplemented with 10% fetal bovine serum (FBS). At 80% confluence, the cells were washed with phosphate buffered saline (PBS) and incubated in medium without FBS for indicated times, respectively. Cells cultured in 10% FBS-medium served as negative control.

### Construct generation

Human XIAP complementary DNA (cDNA) was obtained by RT-PCR of RNA prepared from MCF-7 cells, followed by amplification of the XIAP 3′UTR using a cDNA template and the following primers: forward, 5′-CTACTATAGAGTTAGAGGATCCTTAAG ACATAAAAATTTTTGCTTG-3′ and reverse, 5′-GA TATCTGCGGCCTAGCTAGCTGACGGACCGCGCCC GGTGTCTC-3′. The PCR products were subcloned into NheI- and BamHI-digested pIRESneo3-CMV Vector to obtain the construct XIAP 3′UTR, which was verified by DNA sequencing. The 3′UTR sequence of XIAP and FSCN1 were amplified from the genomic DNA of normal breast tissues and subcloned into the psiCHECK2 dual luciferase reporter plasmid (Promega, USA).

### Establishment of stable cell lines

The cells were seeded at 1 × 10^6^ per 60 mm culture dishes for 24h and then were transiently transfected with 8.0μg pIRES-XIAP 3′UTR or pIRES plasmids using Lipofectamine^TM^ 2000 Transfection Reagent (Invitrogen) per manufacturer's instructions. After 48h, the cells were plated at a low density in medium containing 600 μg/ml geneticin (G418) for MCF-7 cells or 800 μg/ml G418 for MDA-MB-231 cells. Once colonies were formed, individual colonies were isolated and expanded. The cells were continuously maintained in medium containing 300 μg/ml G418 for MCF-7 or 400 μg/ml G418 for MDA-MB-231.

### RNA oligonucleotides and transfection

MiRNAs and small interfering RNAs (siRNAs) were synthesized by GenePharma (Shanghai, China). The miRNA mimics are synthetic duplexes representing mature miRNAs. SiRNA and miRNA transfection was performed using lipo2000 (QIAGEN). 20 nmol/l siRNA or miRNA was used for transfection in serum-free medium. Total RNA and protein were prepared 48 to 72 h after transfection and further used for PCR or western blot analysis.

### RNA isolation, miRNA and mRNA detection

Total RNA, inclusive of the small RNA fraction, was extracted from cultured cells and clinical samples with a mirVana miRNA Isolation Kit (Ambion). Mature miR-29a-5p was reverse-transcribed with specific RT primers, quantified with a TaqMan probe, and normalized by U6 small nuclear RNA using TaqMan miRNA assays (Applied Biosystems). mRNA expression analysis was conducted by quantitative PCR using SYBR green Master MIX (Applied Biosystem), with relative changes calculated by the ΔΔCt method. The sequences of the primers used for qRT-PCR were summarized in Table 1.

**Table 1:** The sequence of the oligonucleotide primers used for real-time PCR experiment

Gene	Sense Strand (5′-3′)	Antisense Strand (5′-3′)
XIAP	CAAGAAATCCATCCATGGCAG	CAGTTAGCCCTCCTCCACAGTG
XIAP 3′UTR-F1/R1	GGCATGTTATGTTGTTCT	AAAGCTCCATTTGTTAAGCCTATCT
XIAP 3′UTR-F2/R2	CTGAGCCAGATCAAAGT	TAACTGCCCTGCCTTCT
FSCN1	CCAGCTATGACGTCTTCCAG	TCGAAGAAGAAGTCCACAGG
LASP1	ATGAACCCCAACTGCGCC	TCAGATGGCCTCCACGTAGTT
E-cadherin	TGCCCAGAAAATGAAAAAGG	GTGTATGTGGCAATGCGTTC
Vimentin	CGAGGAGAGCAGGATTTCTC	GGTATCAACCAGAGGGAGTGA
GAPDH	TGCACCACCAACTGCTTAGC	GGCATGGACTGTGGTCATGAG
miR-29a-5p RT-PCR Primer	GTCGTATCCAGTGCAGGGTCCGAG GTATTCGCACTGGATACGACCTGAAC	
miR-29a-5p qRT-PCR Primer	GCGGCGGACTGATTTCTTTTGGT	ATCCAGTGCAGGGTCCGAGG
U6	GCTTCGGCAGCACATATACTAAAAT	CGCTTCACGAATTTGCGTGTCAT

### Protein extraction and Western blot

Total cellular protein and western blot analysis were performed according to previous studies [43, 44]. The antibodies used were as follows: Dicer (#5362; Cell Signaling), E-cadherin (#610181; BD), FSCN1 (sc-46675; Santa Cruz), GAPDH (sc-365062; Santa Cruz), LASP1 (MAB8991; Merck Millipore), Vimentin (#550513; BD) and XIAP (#14334; Cell Signaling).

### Cell proliferation assay

Cell proliferation was assessed using the Cell-Titer 96 AQueous MTS assay (Promega, Madison, WI) according to the manufacturer's instructions. Briefly, cells were incubated with 200 μl of culture medium in 96-multiwell plates. Media were removed and 200 μl medium containing MTS reagent (10%) was added to each well and incubated at 37°C for 2 h. Then, the absorbance at 570 nm was measured by using a microtiter plate reader (Bio-Rad, CA). The experiments were in triplicate and repeated thrice. The data were summarized as mean ± SD.

### Colony formation assay

Each well of a 6-well culture plate were seeded with 6 × 10^2^ cells and each group contained three wells. After incubation at 37°C for 14 days, the cells were washed twice with PBS and stained with 0.1% crystal violet. The numbers of colonies were counted under a microscope. The experiments were in triplicate and repeated thrice. The data were summarized as mean ± SD.

### Flow cytometry assay

Cell apoptosis was assayed using the Annexin V-Apoptosis Detection kit (BestBio, Shanghai, China) according to the manufacturer's instructions. All the experiments were performed using a FACScalibur cytometer (BD Biosciences, San Jose, CA). Cell cycle distribution was analyzed using the PI method. Each experiment was performed in triplicate and repeated at least once.

### Transwell cell migration and invasion assay

Cell migration and invasion were quantified by trans-well assays using uncoated (8 μm pore size, Corning Costar, USA) filters in 24-well plates. In brief, cells were plated in medium with 0.2% BSA onto the upper chamber of the trans-wells (2 × 10^4^cells/well). Below the insert, the chambers of 24-well plates contained medium supplemented with 10% FBS as the chemoattractant. The chambers were incubated at 37°C with 5% CO_2_. At the end of incubation, cells migrating or invading through the filter to the lower surface were fixed with 4% paraformaldehyde for 30 min and stained with 0.1% crystal violet for 10 min. Migrated or invaded cells were photographed and counted in five randomly chosen fields.

### Wound-healing assay

Tumour cell migration was assessed using a wound-healing assay. In six-well plates, cells were cultured until they reached confluent. After culture in serum-free medium for 24 h, the monolayers were scratched with woundings by plastic tips and then were gently rinsed with PBS three times. Phase-contrast images were captured after further culture for 0, 24 and 48 h.

### Tumor xenograft in nude mice

MCF-7-Vec and MCF-7-XIAP 3′UTR cells (5 × 10^6^ cells per site) were injected into the mammary fat pad of eight 4-week-old BALB/c nude mice (Shanghai Slaccas). Long-release estrogen pellets (Innovative Research of America) were implanted the day before inoculation.

### *In vivo* protocol approval

Research protocols were designed and conducted in accordance with the guidelines set by the Institutional Animal Care and Use Committee, University of Science and Technology of China (USTCACUC1501013).

### Genome-wide transcriptional profiling with microarrays

Gene array was carried out to determine the genes that may have been regulated and involved in the activity of XIAP 3′UTR. Briefly, the cells were washed three times with PBS and suspended in 1.0 ml Trizol reagent (Life Technologies, Inc., Carlsbad, CA, USA). The suspended cells were frozen at −80°C. For microarray analysis, cells in Trizol were shipped on dry ice to KangCheng Bio-Tech (Shanghai, China) for analysis *via* Agilent Whole Human genome Oligo Microarray platform. The RNA preparation and microarray hybridization were performed according to the manufacturer's instructions. Briefly, total RNA from each sample was amplified and transcribed into fluorescent cDNA using the manufacturer's instructions (Agilent's Quick Amp Labeling protocol, version 5.7, Agilent Technologies). The labeled cDNAs were hybridized onto the Whole Human Genome Oligo Microarray (4 × 44 K, Agilent Technologies). After having washed the slides, the arrays were scanned by the Agilent Scanner G2505C. Agilent Feature Extraction software (version 11.0.1.1) was used to analyze the acquired array images. Quantile normalization and subsequent data processing were performed using the GeneSpring GX v11.5.1 software package (Agilent Technologies). After quantile normalization of the raw data, at least 1 out of 4 genes samples have flags in detected (all targets value) were chosen for further data analysis. Differentially expressed genes were identified through fold change filtering. Pathway analysis and GO analysis were applied to determine the roles of these differentially expressed genes had in these biological pathways or GO terms. Finally, hierarchical clustering was performed to show the distinguishable gene expression profiling between samples.

### Immunohistochemistry (IHC)

Formalin-fixed, paraffin-embedded tissue was cut into 4 μm section, de-paraffinized in xylene, rehydrated through graded ethanol, quenched for endogenous peroxidase activity in 3% hydrogen peroxide, and processed for antigen retrieval by heating in 10 mM citrate buffer (pH 6.0) at 90–100°C (XIAP, FSCN1, Ki-67, Bcl-2, Bax, Caspase-3). Sections were incubated at 4°C overnight with XIAP (1:200, Cell Signaling), FSCN1 (1:100, Santa Cruz), Ki-67 (1:400, Cell Signaling), Bcl-2 (1:500, Santa Cruz), Bax 1:500, Santa Cruz) and Caspase-3 (1:200, Santa Cruz). Immunostaining was performed using UltraSensitive S-P Detection Kit (KIT-9720, Maixin, Fuzhou, China), and then color was developed by using a DAB kit (DAB-0031, Maixin, Fuzhou, China). Subsequently, sections were counterstained with hematoxylin. TUNEL assay was performed with an *in situ* cell death detection kit (Roche) according to the manufacturer's instructions. Quantification of immunohistochemical stain intensity was performed as previously described [7, 43].

### Luciferase reporter assay

The full-length 3′UTR of the genes were amplified and cloned downstream of *Renilla luciferase* in a psiCHECK2 vector (Promega). Cells plated on 24-well plates were transfected with 100 ng plasmid and 200 nmol/l miR-29a-5p mimics or negative control. After 48 hours, cells were lysed and assayed with Dual Luciferase Assay (Promega) according to the manufacturer's instructions. Three independent experiments were performed in triplicate.

### Statistical analyses

Data are presented as mean ± SD (standard deviation). Student's *t* test (two tailed) was used to compare two groups, *P* value < 0.05 was considered statistically significant.

### Supplementary data

Supplementary Tables 1, 2, Supplementary Figures 1–6.

## SUPPLEMENTARY MATERIALS FIGURES AND TABLES


