# Supplementary material for: XIAP 3′-untranslated region as a ceRNA promotes FSCN1 function in inducing the progression of breast cancer by binding endogenous miR-29a-5p
Source: Oncotarget. 2017 Feb 7;8(10):16784–800. doi: 10.18632/oncotarget.15159 (PMC5370001; doi:10.18632/oncotarget.15159)
Supplement: Supplementary file 1 [file oncotarget-08-16784-s001.pdf]

XIAP 3′-untranslated region as a ceRNA promotes FSCN1 function in inducing the progression of breast cancer by binding endogenous miR-29a-5p

Supplementary Materials

Supplementary Table 1: Sequence of the oligonucleotides for miRNA and siRNA

Gene	Sense Strand (5′-3′)	Antisense Strand (5′-3′)
Negative control	UUCUCCGAACGUGUCACGUTT	ACGUGACACGUUCGGAGAATT
hsa-miR-29a-5p mimics	ACUGAUUUCUUUUGGUGUUCAG	GAACACCAAAAGAAAUCAGUUU
ASO NC	CAGUACUUUUGUGUAGUACAA	
hsa-miR-29a-5p ASO	CUGAACACCAAAAGAAAUCAGU	
Dicer siRNA	ACAUCAAGGUGCUAAUAGAUU	UCUAUUAGCACCUUGAUGUUU
FSCN1 siRNA	AGCCCUGGGCGUGUAGUGUAA	UUACACUACACGCCCAGGGCU

Supplementary Table 2: List of proteins tested by antibodies and characteristics of the corresponding antibodies used

Protein	Assay	Origin	Dilution	Incubation period
XIAP	WB	#14334; Cell Signaling	1:1000	overnight
XIAP	IHC	#14334; Cell Signaling	1:200	overnight
FSCN1	WB	sc-46675; Santa Cruz	1:500	overnight
FSCN1	IHC	sc-46675; Santa Cruz	1:100	overnight
LASP1	WB	MAB8991; Merck Millipore	1:2000	overnight
Ki-67	IHC	#9449; Cell Signaling	1:400	overnight
Bcl-2	IHC	sc-7382; Santa Cruz	1:500	overnight
Bax	IHC	sc-7480; Santa Cruz	1:500	overnight
Caspase-3	IHC	sc-7272; Santa Cruz	1:200	overnight
E-cadherin	WB	#610181, BD	1:5000	overnight
Vimentin	WB	#550513, BD	1:5000	overnight
Dicer	WB	#5362; Cell Signaling	1:500	overnight
GAPDH	WB	sc-365062; Santa Cruz	1:5000	overnight

Abbreviations: WB, Western blot; IHC, immunohistochemistry;

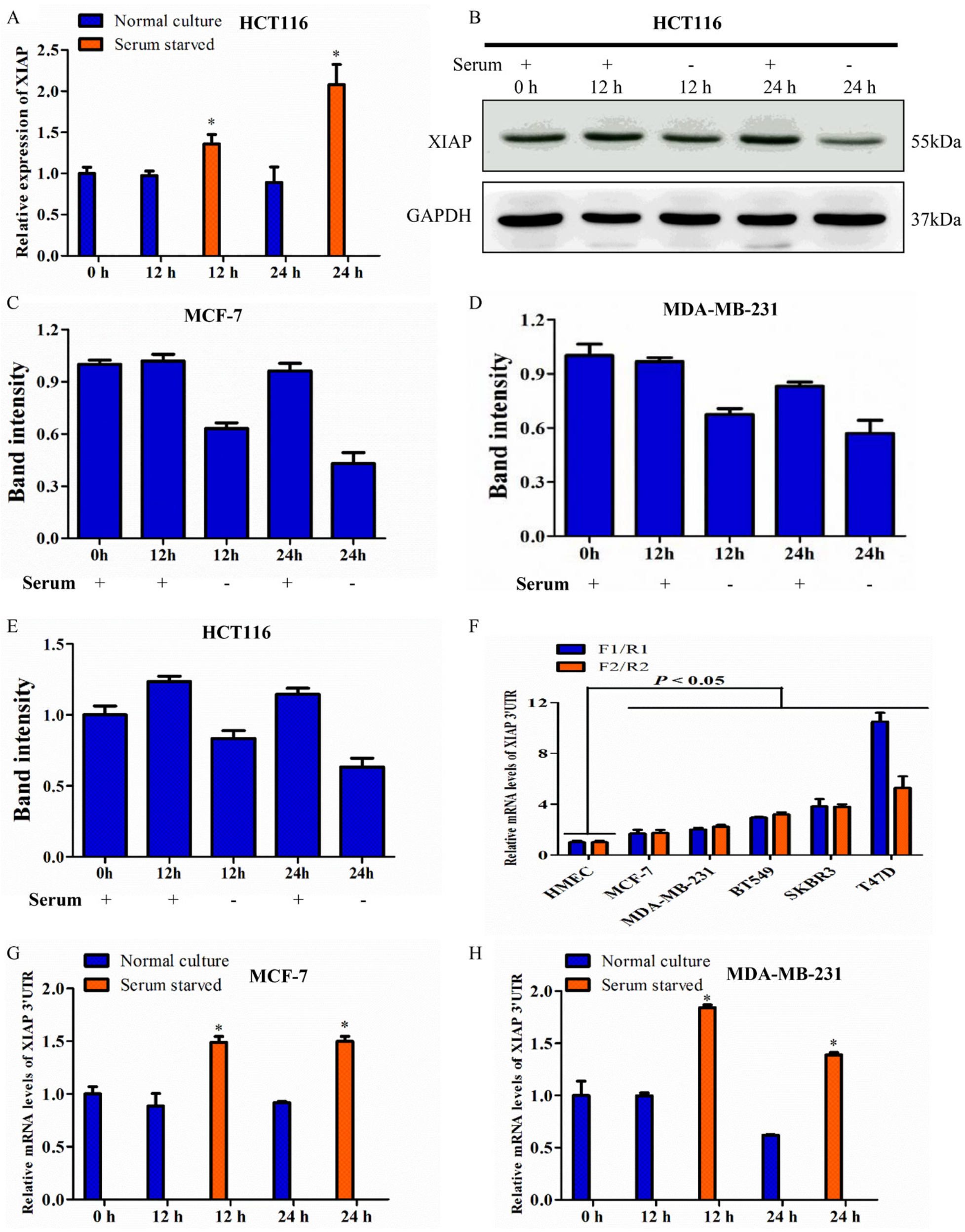

Supplementary Figure 1: Basal expression of XIAP and XIAP 3′UTR in HCT116 and breast cancer cells. (**A–B**) XIAP expression levels in HCT116 cells under the condition of serum deficiency for 12 h or 24 h were detected by qRT-PCR (left) and western blot (right). (**C–D**) The blots shown in Figure 1E and 1G were qualified and the ratio of XIAP to GAPDH was then calculated. (**E**) Corresponding densitometry data of XIAP normalized to GAPDH loading controls in HCT116 cells. The data are represented as mean ± SD from three independent experiments. (**F**) Expression of XIAP 3′UTR in breast cancer cell lines. (**G–H**) Expression of XIAP 3′UTR in MCF-7 (left) and MDA-MB-231 cells (right) under the condition of serum deficiency for 12 h or 24 h were detected by qRT-PCR. *$P < 0.05$.

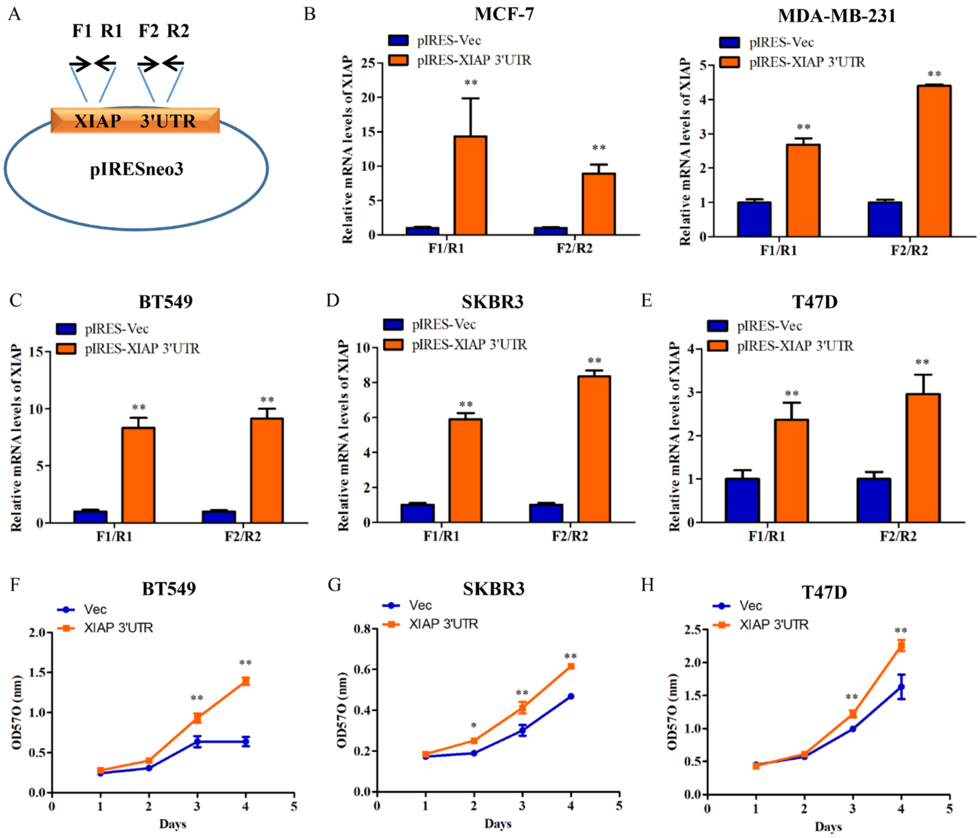

Supplementary Figure 2: Exogenous expression of XIAP 3′UTR increases cell proliferation in breast cancer cells. (**A**) XIAP 3′UTR was cloned and inserted into pIRESneo3 plasmid, producing the XIAP 3′UTR constructs. (**B**–**E**) MCF-7 cells or MDA-MB-231 cells or BT549 cells or SKBR3 cells or T47D cells transfected with XIAP 3′UTR and control vector were subjected to qRT-PCR analyses. (**F**–**H**) Cell viability MTT assays in BT549 cells or SKBR3 cells or T47D cells.*$P < 0.05$, **$P < 0.01$.

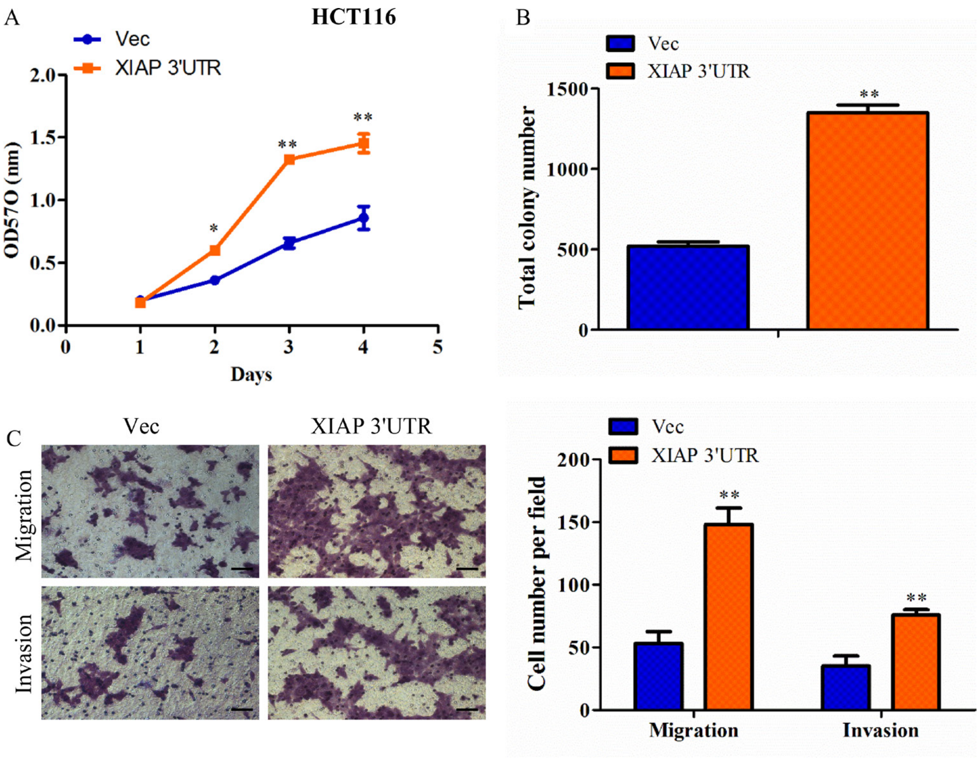

Supplementary Figure 3: XIAP 3′UTR possess oncogenic activity in HCT116 cells similar to breast cancer cells. (**A**) HCT116 cells were transfected with vector or XIAP 3′UTR for 4 days. Cell viability was measured by MTT assay. (**B**) Colony formation assay. (**C**) Transwell migration and invasion assay.

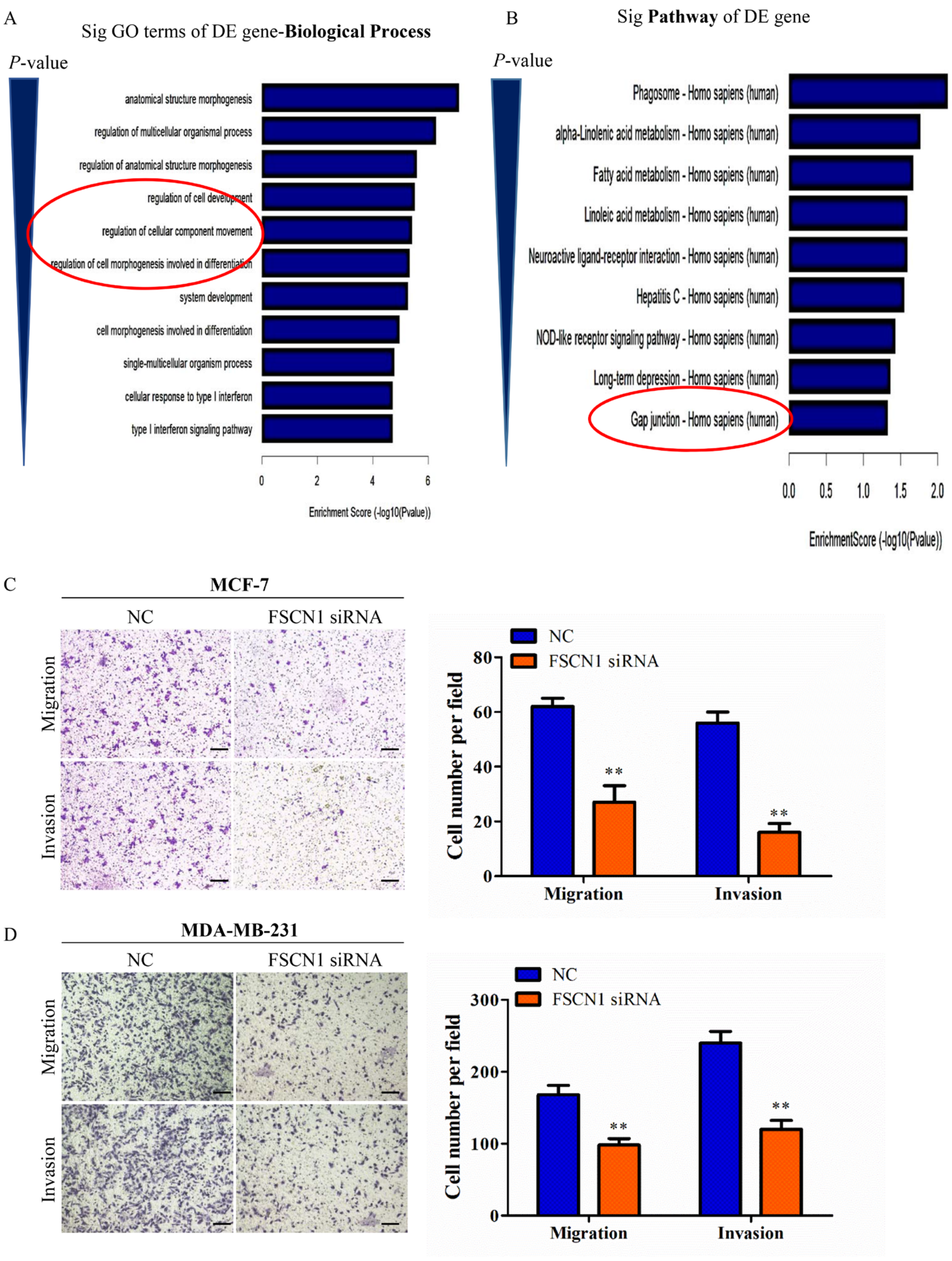

Supplementary Figure 4: Pathways analysis of XIAP 3′UTR to malignant transformation. (**A**) Gene Ontology enrichment analysis of differentially expressed genes between XIAP 3′UTR and vector cells. (**B**) KEGG pathway enrichment analysis of XIAP 3′UTR regulated genes by DAVID software. (**C**–**D**) The effect of FSCN1 expression in cell migration and invasion of MCF-7 and MDA-MB-231, transfected with FSCN1 siRNA or NC, as determined using a transwell assay. Scale bar, 100 μm. **$P < 0.01$.

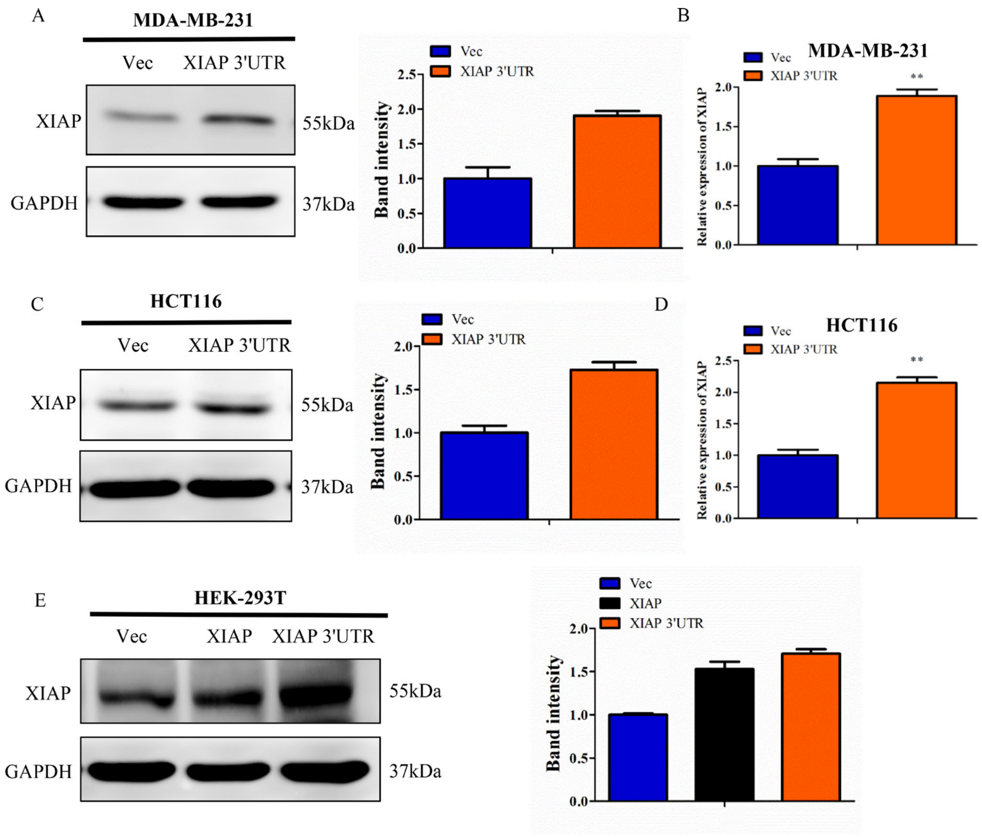

Supplementary Figure 5: Upregulation of XIAP by expression of XIAP 3′UTR. (**A**–**B**) Expression levels of XIAP in MDA-MB-231-XIAP 3′UTR cells were analyzed by western blot (left) and qRT-PCR (right). (**C**–**D**) Expression levels of XIAP in HCT116-XIAP 3′UTR cells were analyzed by western blot (left) and qRT-PCR (right). Representative quantitative data of densitometric analyses were shown in middle. (**E**) XIAP 3′UTR and XIAP coding constructs efficiently expression XIAP protein in HEK-293T cells. Representative quantitative data of densitometric analyses were shown on right. $^{**}P < 0.01$.

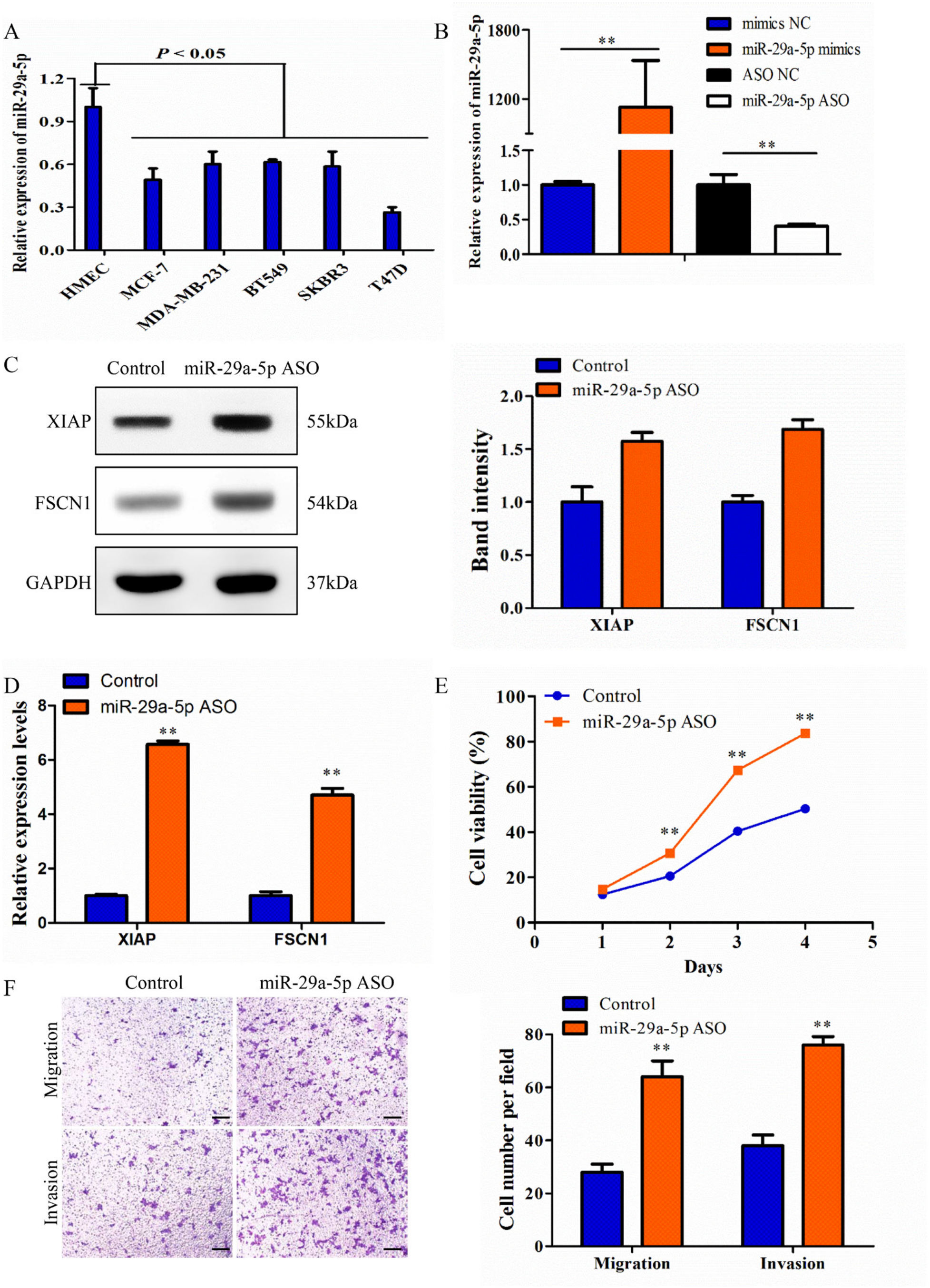

Supplementary Figure 6: Functional analysis of miR-29a-5p in MCF-7 cell line. (**A**) Expression of miR-29a-5p was analysed by qRT-PCR for mRNA levels in breast cancer cell lines. (**B**) Expression of miR-29a-5p in MCF-7 cells transfected with miR-29a-5p mimics or miR-29a-5p ASO and the efficacy was demonstrated by qRT-PCR. (**C**) Lysates of MCF-7 cells transfected with control or miR-29a-5p ASO were analyzed by western blot. Representative quantitative data of densitometric analyses were shown on right. (**D**) Lysates of MCF-7 cells transfected with control or miR-29a-5p ASO were analyzed by qRT-PCR. (**E**) The proliferation rate was measured in cells transfected with miR-29a-5p ASO over a period of 4 days. (**F**) Transwell migration and invasion assay of cells transfected with miR-29a-5p ASO or control. Scale bar, 100 µm. ***P* < 0.01.
